# Spontaneous uterine venous plexus complicated with ovarian rupture in the third trimester of pregnancy: a case report

**DOI:** 10.1186/s12884-023-05556-y

**Published:** 2023-04-13

**Authors:** Jiming Ruan, Gang Zhao

**Affiliations:** Department of Obstetrics and Gynecology, Huzhou Maternity & Child Health Care Hospital, Huzhou, 313000 China

**Keywords:** Third trimester of pregnancy, Spontaneous rupture of the uterine venous plexus, Rupture of ovaries

## Abstract

**Background:**

Spontaneous uterine venous rupture combined with ovarian rupture in late pregnancy is extremely rare. It often has an insidious onset and atypical symptoms, develops rapidly, and is easily misdiagnosed. Wewould like to discuss and share this case of spontaneous uterine venous plexus combined with ovarian rupture in the third trimester of pregnancy with colleagues.

**Case presentation:**

A pregnant woman, G1P0 at 33^+4^ weeks of gestation,was admitted to the hospital due to threatened preterm labour on March 3, 2022. After admission, she was treated with tocolytic inhibitors and foetal lung maturation agents. The patient's symptoms did not improve during the treatment. After many examinations, tests, discussions, a diagnosis, and a caesarean section, the patient was finally diagnosed with atypical pregnancy complicated by spontaneous uterine venous plexus and ovarian rupture.

**Conclusions:**

Spontaneous rupture of the uterine venous plexus combined with ovarian rupture in late pregnancy is an occult and easily misdiagnosed condition, and the consequences are serious. Clinical attention should be given to the disease and prevention attempted to avoid adverse pregnancy outcomes.

## Background

Rupture of the uterine venous plexus is extremely rare in the third trimester, and when combined with ovarian rupture, the odds are negligible. Although the incidence is extremely low, the mortality rate is extremely high. A slight misdiagnosis or a missed diagnosis may lead to maternal and foetal death, making it a serious complication of late pregnancy. The disease usually has an insidious onset, rapid progression, and no clear cause. It is often misdiagnosed as placental abruption, uterine rupture, pregnancy with acute abdomen, etc. If a delayed diagnosis is made in clinical practice, it may threaten the lives of mother and infant and increase mortality. Obstetricians should pay greater attention to this issue.

## Case presentation

The patient, a 24-year-old nulliparous female, had a sudden onset of generalized abdominal pain at 33 + 4 weeks gestation. The patient was admitted to the emergency department of our hospital at 7:43 am on March 03, 2022. The pain was moderate and continuous and had lasted for 5 h before admission. The emergency foetal heart rate was 150 beats per minute, and abdominal palpation and uterine contraction were irregular and of moderate degree. Abdominal ultrasonogram was immediately performed and showed a single intrauterine, live foetus in a head-first presentation and an internal cervix with a U-shaped expansion.The functional cervical length was 11 mm. The working diagnosis at that time was"threatened preterm labour" at 8:27, and the patient was admitted to the obstetrics ward. The physical examination after admission showed a temperature of 36.5°C, heart rate of 100 beats per minute, respiratory rate of 17 breaths per minute, blood pressure of 130/74 mmHg, normal vital parameters, no yellow staining of the skin or sclera, no difference in heart or lung auscultation,no palpable liver or spleen, and no oedema of the lower limbs.The special prenatal examination showed a uterine height of 30 cm, abdominal circumference of 94 cm, foetus in the left occiput anterior (LOA) position, and a foetal heart rate of 140 beats per minute. The patient did not have vaginal bleeding or fluid leakage, had irregular contractions of moderate intensity, and transvaginal examination showed that she was 0.5 cm dilated with a soft cervix. After admission, we communicated with the patient and informed her of her condition. The patient requested continuing the pregnancy;an atosiban intravenous drip was selected to inhibit uterine contractions and 12 mg of betamethasone was given via intramuscular injection to promote foetallung maturation. At 9:23 am, atosiban was given intravenously. During the process of intravenous infusion, irregular contractions could still be felt, but the intensity was weaker than before.At 13:30, the patient still had abdominal pain. Physical examination by the doctor showed tenderness in the abdomen without rebound pain, and irregular contractions of moderate intensity couldbe felt. At 15:18, emergency re-examination by abdominal ultrasound indicated the following: 1. Single foetus in the head-first position, 2. normal umbilical artery blood flow, and 3. ascites (maximum located in the left paracolic sulcus, maximum anteroposterior diameter 60 mm) and flocculent echo in the liver and kidney crypts suggesting the presence of a blood clot (see Fig. 1). Ultrasound suggested that the abdominal effusion could not be excluded as abdominal haemorrhage. Ultrasound-guided abdominal puncture was performed after urgent surgical consultation at 15:22. Dark red bloody fluid was withdrawn, and abdominal haemorrhage was considered. At the same time, ultrasound examination of the liver, gallbladder, pancreas, spleen, kidneys, bladder, and bilateral appendage showed ascites. At 15:35, the multidisciplinary team (MDT) provided a diagnosis and treatment plan. An emergency exploratory laparotomy and caesarean section were performed. During the laparotomy, the volume of bleeding and blood clots in the abdomen was approximately 500 ml. After delivery of the baby, the right ovarian tear was measured at 4*3 cm, and no obvious active bleeding was observed. Along the ovarian tear to the right side of the posterior wall of the uterus, a 10*3 cm parametrial venous plexus was permeated and ruptured, and an obvious active bleeding point was seen within. A 5*2 cm blood clot was observed on the surface of the intestinal tube below the tear, and no obvious active bleeding was observed (see Fig. 2).During the operation, the active bleeding points were ligated in a figure-8 pattern through 3/0 absorbable sutures. After the operation, an abdominal drainage tube was placed, and the volume of total bleeding was 800 ml. The result was G1P1 at 33 + 4 weeks of an LOA preterm live infant after a pregnancy complicated with foetal dystocia and ovarian rupture and uterine venous plexus haemorrhage complicated with pelvic inflammatory disease sequelae. After the operation, the patient was given symptomatic treatment to prevent infection and promote uterine contraction and was discharged 5 days after the operation.

Postoperative pathological examination revealed a parametrial vascular plexus consisting of fibrous smooth muscle tissue with hyperaemia and haemorrhage (see Fig. 3).

### Preoperative auxiliary examination

**Fig. 1:**
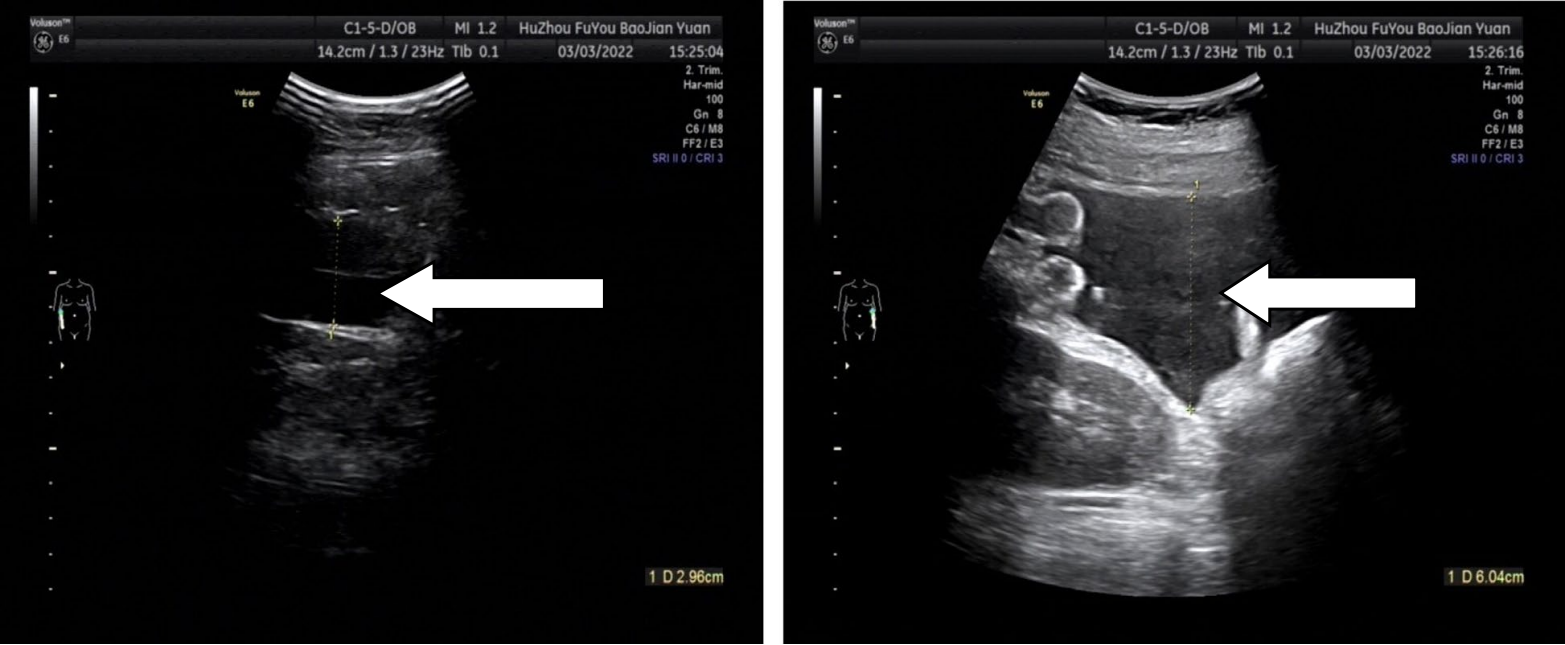
Ultrasonic findings indicating a large amount of peritoneal effusion

### Intraoperative situation

**Fig. 2:**
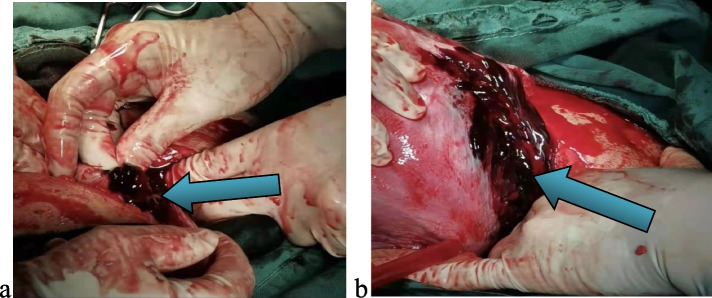
The rupture of the right ovary was 4*3 cm without obvious active bleeding **a**. The uterine venous plexus was 10*3 cm in size, and there was an obvious active bleeding point **b**

### Postoperative pathological results

**Fig. 3:**
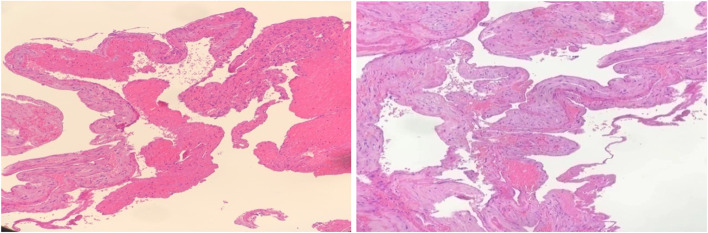
Pathological examination:fibrous smooth muscle tissue (parauterine vascular plexus) with congestion and bleeding

## Discussion and conclusion

### Clinical features and aetiology

The incidence of uterine venous plexus rupture in pregnancy is approximately 1/10000, and fewer than 50 cases have been reported worldwide (see Table 1). The most common torn ligament is the broad ligament of the uterus, accounting for 78.3% of rupture cases. There are very few reports of spontaneous rupture of the uterus [1, 2] and even fewer cases of ovarian rupture. Although the incidence rate is low, there is a high risk of death. According to a research report in the United States conducted 50 years ago, the probability of death for pregnant women caused by abdominal haemorrhage during pregnancy was 49%. More than 50 years later, due to progress in medical treatment, such as advancements in technology, anaesthesia, cardiopulmonary resuscitation, and surgery by obstetricians and gynaecologists, the mortality rate has greatly decreased. At present, the cause of spontaneous uterine venous plexus rupture is still unclear. Hodgkinson and Christiansen [3] stated a number of reasons for this condition. ①Some related muscle activities during pregnancy (such as coughing, defecation, sexual intercourse, and childbirth in the second stage of labour) lead to uterine vascular dilatation and elevated venous pressure, especially in varicose veins of the broad ligament of the uterus, which can be detected during caesarean section [4]. ② During pregnancy, blood volume increases, and uterine arteries and veins are full and varicose. The outer sheaths of the subserosal vein and parauterine vein, which are superficial and thin, have no fascia and lack inherent elasticity, and the small and medium veins lack valves. The enlarged uterus in pregnancy also oppresses the inferior vena cava so that blood flow is blocked, and the venous pressure rises to 2–3 times the normal value. Remaining supine for a prolonged time, severe cough or constipation, sexual intercourse, contractions, and other muscle activities can induce spontaneous rupture of the uterine venous plexus [4–8]. ③ Congenital uterine vascular dysplasia, such as uterine venous malformation, may also be involved. ④ With endometriosis or inflammation in pregnancy, the subserosal and parauterine veins are more superficial and varicose, which makes the blood vessels vulnerable to rupture and bleeding during decidualized endometriosis. A retrospective study by Brosenset et al. [9] found that 90% of the rupture sites of blood vessels were located in the posterior wall and parauterine tissues, and 52% of patients were complicated with endometriosis. Therefore, some scholars believe that endometriosis is the main risk factor for spontaneous rupture of uterine vessels during pregnancy. Xie et al. [10] showed that uterine venous plexus rupture during pregnancy mostly occurs at 32–36 weeks of pregnancy and rarely lasts until full term. However, the probability of pregnancy complicated by ovarian rupture is lower. Zhao et al. [11] and other studies have shown that most ovarian ruptures in pregnancy are ruptures of corpus luteum cysts, which are closely related to the corpus luteum. During pregnancy, the ovary is accompanied by corpus luteum cyst formation, the volume of the ovary increases, the tension of the surface capsule is high, and the texture is brittle. When this is combined with collision or extrusion, spontaneous rupture may occur. Spontaneous ovarian rupture accounts for 58.1% of all ovarian ruptures [12] and occurs on the right side more often than on the left side. At present, no cases of spontaneous ovarian rupture in the third trimester of pregnancy have been reported in the literature, and only when ovarian endometriosis or ovarian abscess is involved may spontaneous ovarian rupture occur.

**Table 1:** Currently reported cases of uterine venous plexus or ovarian rupture in the third trimester of pregnancy worldwide

Cases	Gestational age	Site of haemorrhagic rupture	Inducement	Symptoms	Procedures performed
Miao et al. 2019 [13]	35	Inflammatory oozing of blood into the posterior wall of the uterus	no	Lower abdominal pain	Exploratory laparotomy and caesarean section
Miao et al. 2019 [13]	30	Vessels of the broad ligament of the uterus	no	Lower abdominal pain	Exploratory laparotomy and dissection of the uterus
Miao et al. 2019 [13]	33	Blood vessels in the cornu of the uterus	no	Lower abdominal pain	Exploratory laparotomy and caesarean section
Chen et al. 2003 [14]	31	Blood vessels in the serosal layer of the anterior wall of the uterus	no	Epigastric pain and vomiting	The pregnancy was continuedafter haemostatic suturing
Wu et al. 2003 [15]	35	Posterior wall of the uterus, mesosalpinx, broad ligament vessels	no	Vaginal bleeding	Exploratory laparotomy and caesarean section
Wu et al. 2003 [15]	40	Left uterine venous plexus	no	Lower abdominal pain	Exploratory laparotomy and caesarean section
Li et al. 2019 [16]	40	Blood vessels at the right angle of the anterior wall of the uterus	no	Epigastric pain	Exploratory laparotomy and caesarean section
Yang et al. 2015 [17]	29	A blood vessel emanating from the right posterior wall of the uterus	no	Lower abdominal pain	Exploratory laparotomy and dissection of the uterus
Dai et al. 2013 [18]	39	Subserosal vessels in the anterior wall of the right uterus	no	Epigastric pain and vomiting	Exploratory laparotomy and caesarean section
Dai et al. 2013 [18]	34	Vessels of the proper ligament of the ovary	no	Epigastric pain and vomiting	Exploratory laparotomy and caesarean section
Dai et al. 2013 [18]	38	Vessels of the proper ligament of the ovary	no	Lower abdominal pain	Exploratory laparotomy and caesarean section
Dai et al. 2013 [18]	39	Blood vessels in the isthmus of the posterior wall of the uterus	no	Lower abdominal pain	Exploratory laparotomy and caesarean section
Zhang et al. 1999 [19]	31	Blood vessels in the left posterior wall of the uterus	Lifting heavy objects	Lower abdominal pain	Exploratory laparotomy and dissection of the uterus
Wu C Yet al. 2007 [4]	32	Blood vessels in the posterior wall of the uterus	no	Vaginal bleeding	Exploratory laparotomy and caesarean section
Chiodo Iet al. 2008 [20]	31	Right uterine artery	no	lower abdominal pain and haematuria	Exploratory laparotomy and dissection of the uterus
Hashimoto K et al. 2006 [7]	33	Varicose veins in the posterior wall of the uterus	no	Nausea and vomiting	Exploratory laparotomy and caesarean section
Dubuisson J et al. 2006 [21]	31	The left uterine venous plexus	no	lower abdominal pain	Exploratory laparotomy and caesarean section
Passos Fet al. 2008 [22]	31	Bilateral uterine varices	no	lower abdominal pain	Exploratory laparotomy and caesarean section
Mizumoto Y et al. 1996 [23]	28	Blood vessels in the serosal layer at the base of the uterus	no	Epigastric pain	Exploratory laparotomy and caesarean section
Leung WC et al. 1998 [24]	33	Endometriotic deposits in the right uterine horn	no	Total abdominal pain	Exploratory laparotomy and dissection of the uterus
Renuka T et al. 1998 [25]	36	Ovarian vein	no	lower abdominal pain	Exploratory laparotomy and dissection of the uterus
Katorza E et al. 2007 [26]	29	Vessels of the posterior wall and broad ligament of the uterus	no	lower abdominal pain	Exploratory laparotomy and caesarean section
Ismail KM et al. 1999 [27]	33	Blood vessels in the posterior wall of the uterus	no	lower abdominal pain	Exploratory laparotomy and caesarean section
Roger N et al. 2005 [1]	27	Uterine varices	no	lower abdominal pain	The pregnancy was continued after haemostatic suturing
Inoue T et al. 1992 [28]	29	Uterine varices	no	lower abdominal pain	Exploratory laparotomy and caesarean section
Kalaichandran S et al. 1991 [29]	29	Broad ligament vessels	no	lower abdominal pain	Exploratory laparotomy and caesarean section
Bellucci MJ et al. 1994 [30]	35	Utero-ovarian vessels	Lifting heavy objects	syncope	Exploratory laparotomy and caesarean section
Roche Met al. 2008 [31]	33	Right uterine vessels	no	lower abdominal pain	Exploratory laparotomy and dissection of the uterus
Zhang Y et al. 2009 [32]	29	Right uterine vessels	no	lower abdominal pain	Exploratory laparotomy and dissection of the uterus
Zhang Y et al. 2009 [32]	35	Blood vessels in the posterior wall of the right uterus	no	lower abdominal pain	Exploratory laparotomy and caesarean section
Zhang Y et al. 2009 [32]	30	Vessels in the left uterine horn	no	lower abdominal pain	Exploratory laparotomy and caesarean section
Giulini S et al. 2010 [33]	33	Left broad ligament vessels	no	lower abdominal pain	Exploratory laparotomy and caesarean section
Huisman CMet al. 2010 [34]	36	Vessels of the proper ligament of the ovary	no	lower abdominal pain	Exploratory laparotomy and caesarean section
Nakaya Y et al. 2011 [35]	28	Right uterine varices were present	no	lower abdominal pain	Exploratory laparotomy and dissection of the uterus
Williamson Het al. 2011 [36]	37	Left uterine vein	childbirth	The whole abdomen was tender	Spontaneous delivery after intrauterine foetal death
Al Qahtani NH. 2012 [37]	38	Uterine posterior wall vessels, ovarian vessels	no	lower abdominal pain	Exploratory laparotomy and caesarean section
Munir SI et al. 2012 [38]	38	Left ovarian artery	no	lower abdominal pain	Exploratory laparotomy and caesarean section
Doger E et al. 2013 [39]	32	Right subserosal vessels of the uterus	no	lower abdominal pain	Exploratory laparotomy and caesarean section
Díaz-Murillo R et al. 2014 [40]	37	Vessels in the left uterine horn	no	lower abdominal pain	Exploratory laparotomy and caesarean section
Lim PS et al. 2014 [41]	37	Blood vessels in the posterior wall of the left uterus	no	lower abdominal pain	Exploratory laparotomy and caesarean section
Cozzolino M et al. 2015 [42]	29	Right ovarian vessels	no	lower abdominal pain	Exploratory laparotomy and caesarean section
Zhang Z et al. 2015 [43]	41	Blood vessels in the walls of ovarian cysts	no	lower abdominal pain	Exploratory laparotomy and caesarean section
Petresin J et al. 2016 [44]	28	Venous plexus in the posterior wall of the uterus	no	lower abdominal pain	Exploratory laparotomy and caesarean section
Xie. 2006 [10]	33	The right cornual vein of the uterus	no	lower abdominal pain	Exploratory laparotomy and caesarean section
Xie. 2006 [10]	34	Blood vessels in the left posterior wall of the uterus	no	lower abdominal pain	Exploratory laparotomy and caesarean section
Maya ET et al. 2012 [45]	29	Left fallopian tube vessels	no	lower abdominal pain	The pregnancy was continued after haemostatic suturing
Hamadeh S et al. 2018 [46]	38	Left utero-ovarian venous plexus	no	lower abdominal pain	Exploratory laparotomy and caesarean section

### Differential diagnosis and treatment

In addition to normal labour, the occurrence of lower abdominal pain in the third trimester of pregnancy should also include the possibility of placental abruption and threatened uterine rupture in obstetrics; torsion of ovarian cyst pedicles and rupture of ovarian cysts; surgically acute abdomen with emergency intestinal obstruction, acute gastrointestinal perforation, acute cholecystitis with perforation, acute appendicitis, acute ureteral calculi, etc.; injury and rupture of peripheral organs,including acute bladder rupture, trauma, etc.; and pregnancy complicated by uterine venous plexus rupture or ovarian rupture also needs to be considered. Ultrasound-assisted examination is of great importance for all acute abdomen cases, and sometimes MRI may be needed. In combination with doctors' clinical experience, if other complications are suspected, an emergency consultation should be conducted immediately, all complications should be ruled out, and relevant treatment should be performed in parallel.

In the case of uterine venous plexus complicated with ovarian rupture in the third trimester of pregnancy, after the definite diagnosis of intra-abdominal haemorrhage by ultrasound and abdominal puncture, laparotomy and caesarean section should be performed immediately according to the gestational week. If the pregnancy is in the early stages, we should comprehensively evaluate whether to choose conservative surgical treatment according to the intraoperative situation and the wishes of the family members. Percutaneous uterine vascular embolization may be an alternative and effective obstetric treatment, especially for pregnant women who are not full term, have difficulty becoming pregnant, and have great expectations for the foetus [40].

### Lessons learned

Reviewing this report, there is no clear cause for the situation in this case. Irregular contractions may have been one possible cause, but they were not an absolute cause. After 33 + 4 weeks of gestation, the patient had abdominal pain without any inducement. When the patient went to see a doctor, the emergency doctor only handled the contractions and neglected to perform a physical examination of the abdomen. In addition, emergency ultrasound indicated regression and expansion of the cervix, which was when threatened preterm labour was considered. After admission, the doctor did not check the patient's contractions in depth immediately, and combined with the patient's strong desire to protect the foetus, he gave atosiban, the strongest contraction inhibitor. On the one hand, the contractions were weakened, the abdominal pain was partially relieved, and the condition was easily covered. On the other hand, this approach delayed the detection of the disease and made the disease develop more seriously. If the doctor had not paid enough attention to the patient's complaint during the intravenous drip of atosiban and had not performed a detailed abdominal physical examination again, the consequences would have been more serious and would even have endangered the life of the pregnant woman and the foetus. We must pay attention to the patient's chief complaint, report to the superior doctor in time when the diagnosis is not clear, and conduct relevant examinations, consultations, and even MDT discussions quickly. This case has given obstetricians a lesson to remember: abdominal pain is not a harbinger of labour for all pregnant women. Even if the cervical canal recedes or even if the cervix dilates, we cannot use obstetrics to explain all unexplained abdominal pain. We should always have a sceptical attitude and start from the basics: consultation, calling, discussion, etc., in addition to auxiliary examinations, communication with the patients' families, definite diagnoses in the shortest time possible, and timely treatment. Once again, let us raise basic abdominal physical examinations to new heights and pay attention to them.

## Conclusion

This case is different from previous reports. Most previous reports have been cases of pregnancy complicated with rupture of the uterine blood vessels or veins, and concomitant ovarian rupture has not been reported. Although pregnancy complicated with uterine venous plexus rupture or ovarian rupture is unpredictable, we can prevent these complications by strengthening maternal health care, improving maternal health and prevention awareness, and making future mothers fully aware of the importance of prenatal examinations. Pregnant women should be instructed to avoid sudden increases in intra-abdominal pressure during pregnancy, such as by severe cough and forced defecation, as much as possible. We should also strive to improve the technical level of obstetric teams and summarize their experiences. Clinically, acute persistent abdominal pain in the third trimester of pregnancy should be carefully differentiated.

## Data Availability

All the relevant data are included in the case report. Reasonable requests for any additional data can be obtained by contacting the corresponding author.

## Abbreviations

MDT: Multidisciplinary team
MRI: Magnetic resonance imaging
